# Grand scale genome manipulation via chromosome swapping in *Escherichia coli* programmed by three one megabase chromosomes

**DOI:** 10.1093/nar/gkab298

**Published:** 2021-04-28

**Authors:** Tatsuya Yoneji, Hironobu Fujita, Takahito Mukai, Masayuki Su’etsugu

**Affiliations:** Department of Life Science, College of Science, Rikkyo University, 3-34-1 Nishi-Ikebukuro, Toshima-ku, Tokyo 171-8501, Japan; Department of Life Science, College of Science, Rikkyo University, 3-34-1 Nishi-Ikebukuro, Toshima-ku, Tokyo 171-8501, Japan; Department of Life Science, College of Science, Rikkyo University, 3-34-1 Nishi-Ikebukuro, Toshima-ku, Tokyo 171-8501, Japan; Department of Life Science, College of Science, Rikkyo University, 3-34-1 Nishi-Ikebukuro, Toshima-ku, Tokyo 171-8501, Japan

## Abstract

In bacterial synthetic biology, whole genome transplantation has been achieved only in mycoplasmas that contain a small genome and are competent for foreign genome uptake. In this study, we developed *Escherichia coli* strains programmed by three 1-megabase (Mb) chromosomes by splitting the 3-Mb chromosome of a genome-reduced strain. The first split-chromosome retains the original replication origin (*oriC*) and partitioning (*par*) system. The second one has an *oriC* and the *par* locus from the F plasmid, while the third one has the *ori* and *par* locus of the *Vibrio tubiashii* secondary chromosome. The tripartite-genome cells maintained the rod-shaped form and grew only twice as slowly as their parent, allowing their further genetic engineering. A proportion of these 1-Mb chromosomes were purified as covalently closed supercoiled molecules with a conventional alkaline lysis method and anion exchange columns. Furthermore, the second and third chromosomes could be individually electroporated into competent cells. In contrast, the first split-chromosome was not able to coexist with another chromosome carrying the same origin region. However, it was exchangeable via conjugation between tripartite-genome strains by using different selection markers. We believe that this *E. coli*-based technology has the potential to greatly accelerate synthetic biology and synthetic genomics.

## Graphical Abstract

**Graphical Abstract:**
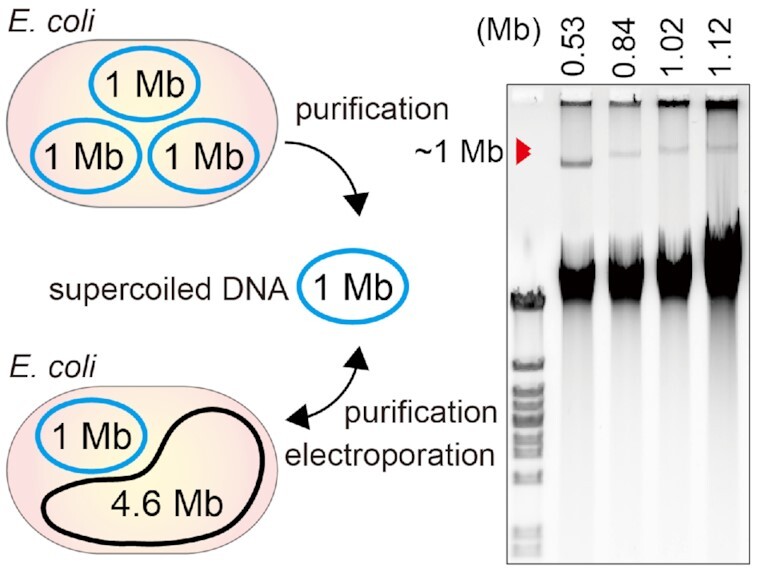
*E. coli* 1-Mb split-chromosomes are suitable for chromosome swapping and implantation.

## INTRODUCTION

Building and booting-up synthetic genomes is a powerful bottom-up approach for understanding and engineering living systems (1–5). To construct bacteria programmed by synthetic genomes, the J. Craig Venter Institute built circular chromosomes in *Saccharomyces cerevisiae* by assembling DNA fragments (1,6–8), and then installed those synthetic genomes into cells of a recipient bacterium that was phylogenetically similar to the species on which the synthetic chromosome was based (1,8,9). To date this genome transplantation (GT) approach has only worked for a small closely related group of Mycoplasma species (5). In those instances, *Mycoplasma capricolum* was converted to a related bacterium such as *Mesoplasma florum* (5,9–11). *M. capricolum* is the only bacterium known to have an extraordinarily high competence for foreign genome uptake (1,9,10,12). On the other hand, *S. cerevisiae* has the potential to maintain a bacterial chromosome as large as 1.8 Mb (5). Whole genomes of mycoplasmas (up to 1.8 Mb) (10), *Acholeplasma laidlawii* (1.5 Mb) (13), *Prochlorococcus marinus* (1.6 Mb) (14), and *Haemophilus influenzae* (1.8 Mb) (15) were cloned into yeast via a cell-fusion method (15). Besides, essential genomes of *Escherichia coli* (16) and *Caulobacter crescentus* (17) were designed and assembled in yeast. Apart from yeast, the 3.5-Mb whole genome of *Synechocystis* PCC6803 was assembled and cloned in the *Bacillus subtilis* chromosome (18). These cloned genomes might be rebooted after their transfer to proper host cells, which should be identical with or closely related to the bacterial species from which the cloned genomes derived. Unfortunately, the straightforward GT approach has not yet been achieved in conventional bacteria such as *E. coli*.

The *E. coli* genome has been engineered by genome segment swap methods (19–24). Instead of transforming *E. coli* directly with a synthetic genome, synthetic segments as large as 100 kb replaced the original genomic regions via recombination and were assembled into a fully synthetic genome via conjugation (19,22,23). These sequential and hierarchical assembly methods successfully produced *E. coli* strains having a different genetic code (19,22). One drawback of this approach is that the genomic structures remained unchanged from their template genomes. To achieve more flexible genome design, Wang *et al.* developed a refined method for chromosome fission and fusion and demonstrated the transfer of a genomic region to another position in the genome (25). Despite these innovations, there is a need for more straightforward methods for flexible genome design and for rebooting a heterologous genome in *E. coli* (16).

**Figure 1.**
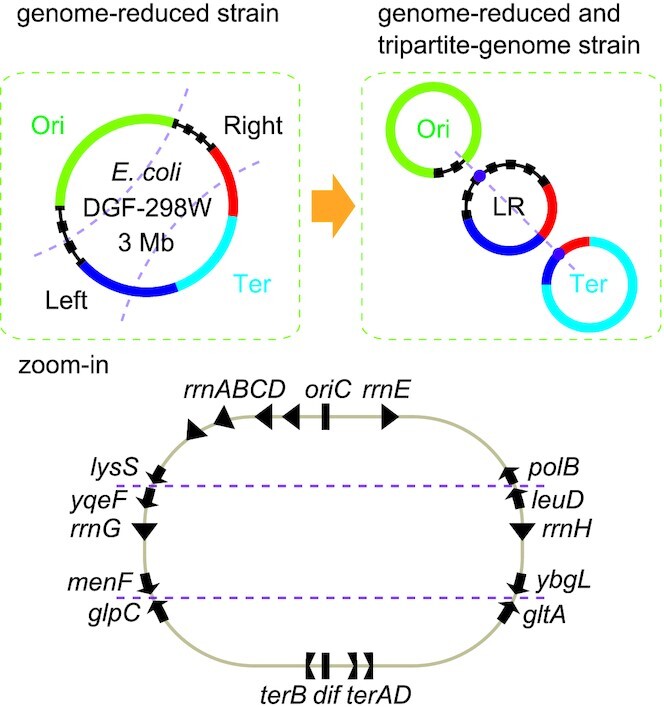
Design principle of tripartite genomes. In the zoom-in view, the ribosome RNA operons (*rrnABCDEHG*), *oriC* and *dif*, the *ter* sequences, and the eight genes at the borders are indicated.

One approach is the downsizing of the *E. coli* genome from 4.6 Mb (26–29). Among the several genome-reduced strains of *E. coli*, DGF-298W (29) carries the second smallest genome (2.98 Mb), grows vigorously, and accepts various types of genetic engineering. However, the 3-Mb genome is still three times larger than the *Mycoplasma mycoides* genome (1.08 Mb) that is used in GT reactions (1). A compromising way of downsizing is to split the single chromosome genome. Several strains of *E. coli* with two chromosomes have been established by using an additional pair of replication origins (*ori*) and partitioning loci (*par*) for the secondary chromosome (25,30–32). During the establishment of the two-chromosome strains of DGF-298W (2 Mb and 1 Mb) (32), we realized that chromosomes of up to 1-Mb in size can be handled *in vitro* as covalently closed supercoiled molecules and that 0.5-Mb supercoiled chromosomes can be even purified using a commercial DNA extraction kit (32). Furthermore, a previous study reported that up to 0.5-Mb synthetic chromosomes can be electroporated into *E. coli* competent cells (33). These findings tempted us to establish a DGF-298W-derived strain consisting of three 1-Mb split-chromosomes (Figure 1) and to assess whether each split-chromosome can be electroporated into *E. coli* cells.

In the present study, we first identified a good combination of *ori-par* systems that enabled stable maintenance of three split-chromosomes in *E. coli*. The 3-Mb chromosome of DGF-298W was safely split into three 1-Mb chromosomes (1.12, 0.84 and 1.02 Mb). Next, the transferability of each split-chromosome was examined via electroporation or via conjugation. The 0.84-Mb and 1.02-Mb chromosomes were purified, electroporated into commercial competent cells of *E. coli* HST08, and extracted again from the transformed cells. The 1.12-Mb chromosome was successfully purified but was not introduced via electroporation. Upon conjugation, the 1.12-Mb chromosome transferred from the donor strain replaced the 1.12-Mb chromosome in the recipient strain. Thus, we demonstrate chromosome swapping via conjugation (25) and chromosome implantation via electroporation in the 1/3 genome-scale in *E. coli*.

## MATERIALS AND METHODS

### Agarose gel electrophoresis analyses

The pulsed-field gel electrophoresis (PFGE) analysis of chromosomes was performed in the same manner as before (32). In short, the CHEF-DR II and III Pulsed Field Electrophoresis Systems (Bio-Rad) were used for resolving DNA molecules embedded in agarose plugs. The DNA size markers used were *S. cerevisiae* Chromosomes and *Hansenula wingei* Chromosomes (Bio-Rad). The canonical agarose gel electrophoresis analysis of chromosomes was performed using 0.5% gels and 0.5× TBE buffer in the same manner as before (32). The size marker used was Marker 3 (NIPPON GENE). In both cases, the gels were stained with dsGreen, a SYBR GREEN-I analogue, (Funakoshi) and scanned by using a Typhoon FLA 9500 (GE Healthcare).

### BAC Purification and electroporation

Chromosomes were purified by using NucleoBond Xtra BAC kit (Takara Bio) essentially as described before (32) but with a few modifications. Cells were harvested from 750 ml of LB cultures incubated overnight at 37°C using an INNOVA 42 (Eppendorf). Cell extracts from cells corresponding to OD_600_ = 1500 at maximum were applied to each column of anion exchange resin. After elution of DNA from a column by using 15 ml of Buffer ELU-BAC, the eluate was transferred to six 5-ml Eppendorf tubes and subjected to isopropanol precipitation by using 1 μl of Ethachinmate (NIPPON GENE) as a carrier solution. After the isopropanol precipitation step, the DNA pellets were air-dried for 15 min and dissolved with 83 μl of TE buffer (pH 8.0) (NIPPON GENE) overnight in the refrigerator. The eluate was transferred to a new Lo-bind tube by using a wide bore tip and stored in the refrigerator. For electroporation, a 2 μl aliquot of purified chromosome solutions was mixed with 50 μl of *E. coli* HST08 Premium Electro-Cells (Takara Bio) by pipetting five times using a wide bore tip. After incubation on ice for 1 minute, the cell-DNA mixture was transferred into a 0.1 cm gap electroporation cuvette (Bio-Rad), and electroporated by using an ELEPO21 (NEPA GENE). The condition of electroporation was set as follows; poring pulse (voltage: 1600 V, pulse width: 3.5 ms, pulse interval: 50.0 ms, number of pulses: 1, polarity: +) and transfer pulse (voltage: 100 V, pulse width: 50.0 ms, pulse interval: 50.0 ms, number of pulses: 3, polarity: +/–). Four vials of HST08 competent cells were used for electroporating each chromosome to obtain enough number of positive colonies.

### Plasmid construction and cell engineering

Plasmids were developed by using PrimeStar Max (Takara Bio) and KOD One Master Mix (TOYOBO) for PCR, In-Fusion (Clontech) for *in vitro* assembly, and chemical competent cells of HST08/Stellar cells (Takara/Clontech) for transformation. Some of the plasmids and BAC vectors were developed previously (32), and the others were newly developed by local modifications (see Table 1). Some of the DNA cassettes used for the λ Red recombination were developed previously (32), while the others were newly prepared. The information for new DNAs is provided as supplementary. The plasmid and chromosome maps and sequences were made using SnapGene software. The sequence files of 1-Mb chromosomes were made using the nearly-complete draft genome sequence data of DGF-298W (32). The λ Red recombination was performed essentially as described previously (32) but with a new helper plasmid mediating the recombination. The new helper plasmid encodes the *recA* gene in addition to the λ *bet-exo* genes on pMW118 under control of the arabinose promoter, because *recA*-deficient derivatives of DGF-298W were used in this study. The antibiotic concentrations were 100 μg/ml for carbenicillin, 15, 25, 30 μg/ml for kanamycin, 17, 25, 34 μg/ml for chloramphenicol, 100 μg/ml for spectinomycin, Zeocin, and Blasticidin S, 5, 7, 10 μg/ml for tetracycline, 3.5 or 7 μg/ml for gentamicin, and 50 or 100 μg/ml for hygromycin. The colony-direct PCR was performed by using GoTaq Green Master Mix (Promega). The Flp-POP cloning was performed as described previously (32), but with a choice of two helper plasmids carrying either of the spectinomycin and gentamicin selection markers. l-Arabinose was added to growth media to induce the expression of flippase and HK022 phage integrase, as previously described (32). For the self-circularization of the 1.12-Mb region, two *FRT* cassettes were inserted between the *polB* gene and the *leuD* gene and between the *lysS* gene and the *yqeF* gene. The conjugal transfer of chromosomes was performed in the same manner as before by using the helper plasmid pBAD-traRP4min (32). l-Arabinose was added to growth media to induce the expression of the conjugation apparatus, as previously described (32). A strain list is provided as Table 2.

**Table 1.** Plasmids, BAC vectors and chromosomes used in this study

name	Description
pMW118-Aba	Helper plasmid for λ-red recombination with *recA* and λ *bet-exo* cloned under *araC*-Para*BAD*
pMW118spec-flp-int*	Helper plasmid for Flp-POP cloning with flippase and HK022 *int* cloned under *araC*-Para*BAD*
pMW118gent-flp-int	pMW118spec-flp-int derivative with a *gent* gene
pBAD-traRP4min*	Helper plasmid for RP4 *tra* expression, Δ*oriT*
pPKOZ-attB*	BAC vector composed of *oriC*, *sopABC*, *kan*, *attB*
pPKOZ-attB-spec	BAC vector derived from pPKOZ-attB with a *spec* gene
pVtu9xT*	BAC vector composed of *V. tubiashii ori2-par2*, *kan*, *attB*, *oriT*
pVtu9xT-PEM7-spec	BAC vector derived from pVtu9xT with a replacement of *kan* by *spec*
pVtu9xF	BAC vector composed of *V. tubiashii ori2-par2*, *kan*, *attB*, a remnant *FRT*
Chr^LR^ (RGF152/YST01)	Chr^LR^ (pPKOZ-attB-spec) purified from RGF152 or YST01 The cloned regions: from *leuD* to *ybgL* through *rrnH* and from *yqeF* to *menF* through *rrnG*. The selection markers: kanamycin, spectinomycin, and tetracycline
Chr^Ter^ (HF053)	Chr^Ter^ (pVtu9xF) purified from HF053 The cloned region: from *gltA* to *glpC* through *dif* The selection marker: kanamycin
Chr^Ori^ (HF054)	Chr^Ori^ purified from HF054 The cloned region: from *lysS* to *polB* through *oriC* with *endA::tet* The selection markers: tetracycline, hygromycin, chloramphenicol, and Zeocin

*These plasmids were reported previously (32).

**Table 2.** *E. coli* strains used in this study

Name	Description
DGF-298W	DGF-298WΔ100::revΔ234::SC (a gift from KHK collection)
RGF008C*	DGF-298W Δ*sacB-cat* Δ*terFIJ* Δ*recAX* (renamed RGF008 Δ*recAX*)
RGF093*	RGF008C-derived, [Chr^Ori+LR^ & Chr^Ter^ (pVtu9xT)]
RGF094*	RGF008C-derived, [Chr^Ori+LR^ & Chr^Ter^ (pPKOZ-attB)]
RGF123	RGF008C-derived, [Chr^Ori^ & Chr^LR+Ter^ (pVtu9xT)]
RGF124	RGF008C-derived, [Chr^Ori^ & Chr^LR+Ter^ (pPKOZ-attB)]
RGF108	RGF093 derivative with *hyg-cat-zeo::dif2v2-tet* on Chr^Ori+LR^
RGF109	RGF094 derivative with *hyg-cat-zeo::dif2v2-tet* on Chr^Ori+LR^
RGF138	RGF108-derived, [Chr^Ori^ & Chr^LR^ (pPKOZ-attB-spec) & Chr^Ter^]
RGF140	RGF109-derived, [Chr^Ori^ & Chr^LR^ (pVtu9xT-PEM7-spec) & Chr^Ter^]
RGF147	RGF138 derivative with *hyg-cat-zeo::dif201-bsd-bsd* on Chr^Ori^
RGF152	RGF147 derivative with *endA::oriT-zeo* on Chr^Ori^
RGF156	RGF152 transformed with pBAD-traRP4min
RGF159	RGF152 derivative with *zeo::hyg* on Chr^Ori^ and with *dif::dif2N2-cat* on Chr^Ter^
RGF160	RGF159 derivative with Chr^Ori^ transferred from RGF152 via conjugation
RGF161	RGF159 transformed with pBAD-traRP4min
RGF162	RGF152 derivative with Chr^Ter^ transferred from RGF159 via conjugation
HF026	HST08 *tus::zeo* carrying a 530-kb BAC
HF033	RGF008C derivative with *endA::tet*
HF053	HF033-derived, [Chr^Ori+LR^ & Chr^Ter^ (pVtu9xF)]
HF054	HF033-derived, [Chr^Ori^ & Chr^LR+Ter^ (pVtu9xF)]
YST01	HST08 carrying Chr^LR^ originated from RGF152
YST03	HST08 carrying Chr^Ter^ originated from HF053

*These strains were reported previously (32). The *dif* variant sequences are as follows; *dif2N2*: GGTGCGCATAATATGTGTTATGTTAAAT, *dif201*: GGTGCGCATAATCATGATTATGTTAAAT, *dif2V2*: AGTGCGCATTACGTGCGTTATGTTAAAT.

### Phenotyping of tripartite-genome strains

The growth rates of the wildtype DGF-298W strain and two lines of tripartite-genome strains RGF138 and RGF140 in antibiotic-free LB media in L-shaped tubes were measured in an automatic manner by using a compact rocking incubator TVS062CA (Advantec). The growth condition was set as follows; temperature: 37°C, shaking speed: 60 rpm, measurement while shaking, waiting time for measurement: 10 s, measurement interval: 10 min. The fluorescence microscope observation of the three strains was performed by using a ZEISS Axio Observer (ZEISS) in the dark room. The cells were cultured in antibiotic-free LB media in test tubes at 37°C until reaching OD_600_ = 0.2. Samples were prepared by mixing 5 μl of the fresh cell cultures and 5 μl of 10 μg/ml DAPI in methanol and incubated for 4 min in the dark. Samples were observed by an agar pad method. A pad of 1.5% STAR Agar L-grade 01 (RIKAKEN) was made on a slide glass by sandwiching between two slide glasses using vinyl tape as spacers. A 0.5 μl aliquot of samples was placed on the agar pad, incubated for 5 min in the dark at room temperature, and covered with a cover glass. The sample images were taken under 100× Oil objective for TL Phase and DAPI, and overlaid using NIH ImageJ 1.52k. Cell lengths were measured using ImageJ with a set scale (distance in pixels: 1, known distance: 0.063, Pixel aspect ratio: 1.0, unit of length: μm). The bacterial viability test was performed using SYTO 9 and propidium iodide (PI). DGF-298W, RGF138, and RGF140 cells were cultured in antibiotic-free LB media in test tubes with vigorous shaking at 37°C. Cells in the exponential growth phase (OD_600_ = 0.3) were fixed and stained for live/dead microscopy.

## RESULTS

### Design principle of tripartite genomes

For splitting the DGF-298W genome, we needed three *ori-par* systems that can be used simultaneously for the replication and partitioning of three split-chromosomes. While the *E. coli* genome carries a typical origin of replication (*oriC*), the chromosome partitioning mechanism is not yet fully understood but is different from other bacteria utilizing the typical *parABS* system (34). For generating a sub-chromosome in *E. coli*, two types of *par* systems have been used. One is the *par* locus of the *E. coli* F plasmid (35), or *sopABC*, while the other is the *parABS2* system derived from the *ori* and *par* locus of the *Vibrio* secondary chromosome (the *ori2-par2* locus) (31). Recently, we demonstrated that a 1.02-Mb region popped-out from the 2.98-Mb genome of DGF-298W can be maintained when fused with either a *sopABC-oriC* pair or the *ori2-par2* system of *Vibrio tubiashii* (32). This meant that we have at least two reliable *par* systems and that the 1.96-Mb chromosome was controlled by the native chromosome partitioning mechanism.

Another thing to be considered is chromosome structuring (36) such as the macrodomain structure and the chromosome symmetry. It is known that the genome chromosome of *E. coli* is organized into multiple macrodomains. The Ori macrodomain includes the *oriC* locus, most of the ribosomal RNA operons, and the *migS* (30) and *maoSP* loci (37), which are considered as important for the partitioning and organization of this macrodomain. The Ter macrodomain is organized by the MatP/*matS* system (38) and includes the chromosome dimer resolution site (*dif*) (39). For simplicity, the two other macrodomains located in the left and right sides of the genome were defined as Left and Right macrodomains in this study, respectively. The directions of highly transcribed genes and regulatory DNA motifs (such as KOPS and *ter* motifs) along the Ori-Ter axis are also important for replication fork processivity and for chromosome segregation (Figure 1) (36). In particular, the position and direction of Tus-*ter* replication fork traps is critical (25,32,36,40). Considering all these things, a safe design principle of tripartite genomes may be individual circularization of the Ori and Ter macrodomains and joining of the Left and Right macrodomains (Figure 1). In this study, we define the Ori, Ter, and Left+Right chromosomes Chr^Ori^, Chr^Ter^ and Chr^LR^, respectively.

### Circularization of the Ori macrodomain

**Figure 2.**
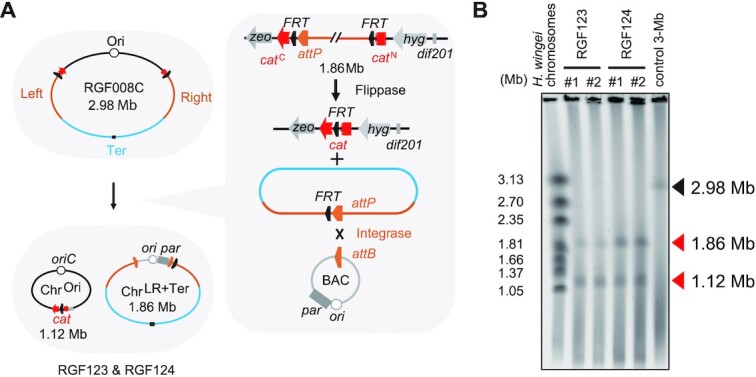
Circularization of the Ori macrodomain by Flp-POP cloning. (**A**) Scheme for the pop-out of Chr^LR+Ter^ by Flp-POP cloning and the concurrent generation of Chr^Ori^. The resultant bipartite-genome strains gained resistance to chloramphenicol via the fused resistance gene. Two types of BAC vectors, pVtu9xT and pPKOZ-attB, were used to develop bipartite-genome strains RGF123 and RGF124, respectively. (**B**) PFGE analysis of the chromosomes of two colonies of RGF123 [Chr^Ori^ & Chr^LR+Ter^ (pVtu9xT)] and two colonies of RGF124 [Chr^Ori^ & Chr^LR+Ter^ (pPKOZ-attB)]. The DNA size markers used for PFGE are *Hansenula wingei* chromosomes.

A 1.12-Mb region which roughly corresponds to the Ori macrodomain was circularized to assess whether this region alone can be stably maintained by the native chromosome partitioning mechanism. For the pop-out of the remaining 1.86 region, our Flp-POP cloning method (32) was employed (Figure 2A). In short, a genomic region flanked by two *FRT* recombination sites was excised using flippase and fused with a bacterial artificial chromosome (BAC) vector by a phage integrase in a site-specific manner. The flippase-*FRT* recombination regenerates a full-length chloramphenicol acetyltransferase gene (*cat*) and thus confers chloramphenicol (Cm) resistance to cells. The pVtu9xT or pPKOZ-attB BAC vectors employ the *V. tubiashii ori2-par2* system and the *oriC*-*sop* pair, respectively. After induction of the flippase and the phage integrase, recombinant cells were selected on Cm-containing agar plates. Two Cm-resistant colonies for each BAC vector were analysed by pulsed-field gel electrophoresis (PFGE) and confirmed to have two chromosomes of 1.12-Mb (Chr^Ori^) and 1.86-Mb (Chr^LR+Ter^) (Figure 2B). Thus, two types of bipartite-genome strains, RGF123 and RGF124, were established (Table 2). This result showed that the 1.12-Mb Chr^Ori^ was stably maintained without the aid of any additional *par* system.

### Developing tripartite-genome strains

**Figure 3.**
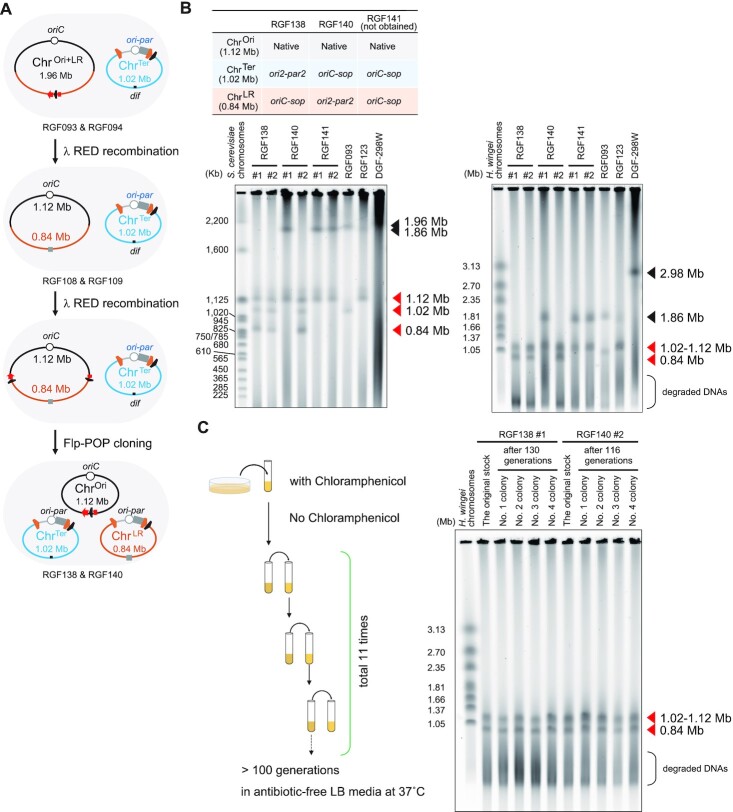
Development and establishment of tripartite-genome strains. (**A**) Scheme for the development of tripartite-genome strains from bipartite-genome strains. (**B**) PFGE analyses of split-chromosomes from tripartite-genome strains. For each combination of BAC vectors shown in the table, two Cm-resistant colonies were chosen and analysed by PFGE. The same samples were analysed in two different PFGE conditions; the chromosomes were resolved by using 0.5% gel and 0.5x TBE buffer to separate DNA bands around 1-Mb (upper left gel) or 0.8% gel and 1× TAE buffer to separate DNA bands around 2-Mb (upper right gel). RGF093 with 1.02-Mb Chr^Ter^ and 1.96-Mb Chr^Ori+LR^ and RGF123 with 1.12-Mb Chr^Ori^ and 1.86-Mb Chr^LR+Ter^ were used as controls. (**C**) PFGE analysis of the three-chromosome configurations after >100 generations. Descendants of both RGF138 and RGF140 strains maintained their tripartite-genome configuration even after >100 generations without antibiotics. The chromosomes were resolved by using 0.8% gel and 1× TAE buffer (lower gel). The absence of fused chromosomes means that the three-chromosome configuration was maintained.

Tripartite-genome strains were developed from bipartite-genome strains by repeatedly performing the Flp-POP cloning (Figure 3A). In our previous work (32), we developed bipartite-genome strains carrying two chromosomes of 1.96-Mb (Chr^Ori+LR^) and 1.02-Mb (Chr^Ter^). The 1.02-Mb region including the Ter macrodomain had been popped-out from the 2.98-Mb genome by using either of the pVtu9xT and pPKOZ-attB BAC vectors to establish RGF093 and RGF094, respectively (Table 2). To perform the second Flp-POP step, the *cat* marker generated by the first Flp-POP step was removed from the 1.96-Mb Chr^Ori+LR^ by λ Red recombination using a tetracycline resistance gene (*tet*) (Figure 3A). Then, the split *cat* cassettes were newly inserted into the Chr^Ori+LR^ by λ Red recombination (Figure 3A). The BAC vectors for the second Flp-POP cloning, pPKOZ-attB-spec and pVtu9xT-PEM7-spec, encoded a spectinomycin resistance gene (*spec*) in addition to the *kan* gene or instead of the *kan* gene, respectively. From the 1.96-Mb Chr^Ori+LR^, the pop-out of the 0.84-Mb region corresponding to the Left and Right macrodomains was attempted by using three combinations of BAC vectors (Figure 3B). First, *ori2-par2* and *oriC-sop* were used for Chr^Ter^ and Chr^LR^, respectively. Second, *oriC-sop* and *ori2-par2* were used for Chr^Ter^ and Chr^LR^, respectively. Lastly, *oriC-sop* was repeatedly used for both Chr^Ter^ and Chr^LR^ chromosomes. Two Cm-resistant colonies for each BAC vector combination were analysed by PFGE (Figure 3B). Both colonies had three split-chromosomes when their Chr^Ter^ and Chr^LR^ were powered by *ori2-par2* and *oriC-sop*, respectively. This tripartite-genome strain was named RGF138. When the opposite BAC combination was used, one of the two colonies had three split-chromosomes, while the other had Chr^Ori^ and Chr^LR+Ter^, which may have emerged by the spontaneous fusion of the Chr^Ter^ and the excised 0.84-Mb region. The former tripartite-genome strain was named RGF140. In contrast, both colonies had Chr^Ori^ and Chr^LR+Ter^ when the *oriC-sop* type BAC vectors were used repeatedly, suggesting that two different types of BAC vectors should be used. Next, the stability of the three-chromosome configurations of the RGF138 and RGF140 strains was investigated by performing another PFGE analysis after >100 generations without antibiotics (Figure 3C). Each of the four sub-strains examined for each strain maintained the three-chromosome configuration (Figure 3C), leading to the conclusion that two kinds of tripartite-genome strains were successfully established.

### Properties of the tripartite-genome strains

**Figure 4.**
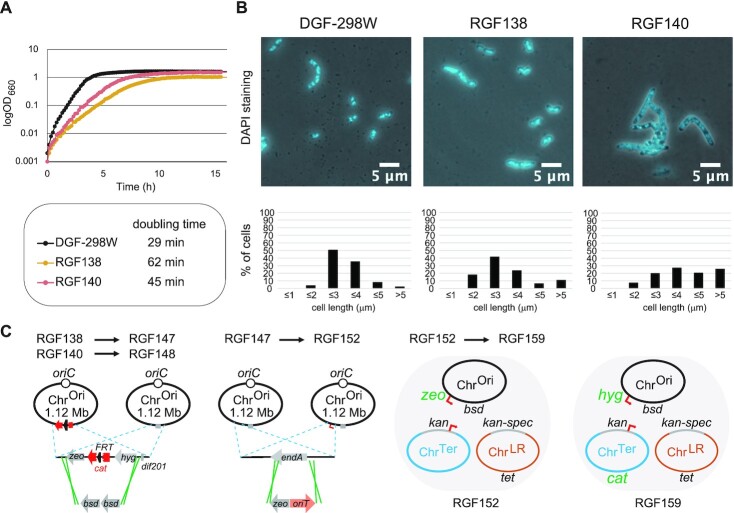
Phenotypes of tripartite-genome strains. (**A**) Growth of tripartite-genome strains. Cells were incubated at 37°C with shaking (60 rpm) in antibiotic-free LB media using L-shaped tubes. Doubling times were computed from the growth curve data. (**B**) Overlay of phase contrast and DAPI stained microscopy images of tripartite-genome strains (upper panels) and distribution of their cell lengths (lower panels). Exponential phase cells growing at 37°C in antibiotic-free LB media were observed and counted. The distribution of cell lengths from 210 DGF-298W cells, 239 RGF138 cells, and 203 RGF140 cells are shown below each picture. (**C**) A diagram depicting the genetic modifications to RGF138 and RGF140.

The growth profiles and cell shapes of RGF138 and RGF140 were investigated. In an LB growth medium in an L-shaped tube with a vigorous shaking, RGF138 and RGF140 grew about 2 times and 1.5 times more slowly than their parent DGF-298W, respectively (Figure 4A). This result is expected since these strains lack any sophisticated mechanism of regulating and synchronizing the replication and segregation of three split-chromosomes. Next, the cell shapes of RGF138, RGF140 and DGF-298W were observed using microscopy (Figure 4B). Interestingly, DGF-298W and RGF138 tend to have a typical rod-shaped morphology, while a large proportion of RGF140 cells were filamentous. Filamentous cells were often observed in the population of our bipartite-genome strains, probably due to the uncontrolled replication and segregation of the chromosomes. A live/dead microscopy assay indicated that the viabilities of DGF-298W and RGF138 in the exponential growth phase were not significantly different (80% versus 78%), whereas only 53% of the RGF140 cells or filaments were viable.

RGF138 and RGF140 were subjected to further genetic engineering (Figure 4C and Table 2). First, they were engineered to eliminate the *cat* gene on Chr^Ori^ by λ Red recombination using two copies of Blasticidin S deaminase genes (*bsd-bsd*), to develop RGF147 and RGF148, respectively. By an unknown reason, RGF140 was highly resistant to the genetic engineering, while RGF138 accepted it. Altogether, RGF138 appeared to have retained wildtype phenotypes in terms of the usability, at the expense of growth rate, and was chosen as the tripartite-genome strain used for further studies. Derivatives of the RGF138 strain was developed by repeating λ Red recombination (Figure 4C). The *endA* gene on the Chr^Ori^ of RGF147 was replaced by a Zeocin resistance gene (*zeo*) together with an *oriT* sequence, to develop RGF152. Thus, in RGF152, the EndA endonuclease, which degrades DNA during purification (41), is missing, and the Chr^Ori^ and Chr^Ter^ are each equipped with the *oriT* sequence of the RP4 plasmid for enabling their individual conjugal transfer using a helper plasmid pBAD-traRP4min (32). Furthermore, the *dif* sequence on the Chr^Ter^ of RGF152 was replaced by a *cat* gene together with a *dif* variant sequence *dif2N2* (Table 2), and the *zeo* gene at the *endA* locus was replaced by a hygromycin resistance gene (*hyg*), to develop RGF159.

### Purification and electroporation of split-chromosomes

**Figure 5.**
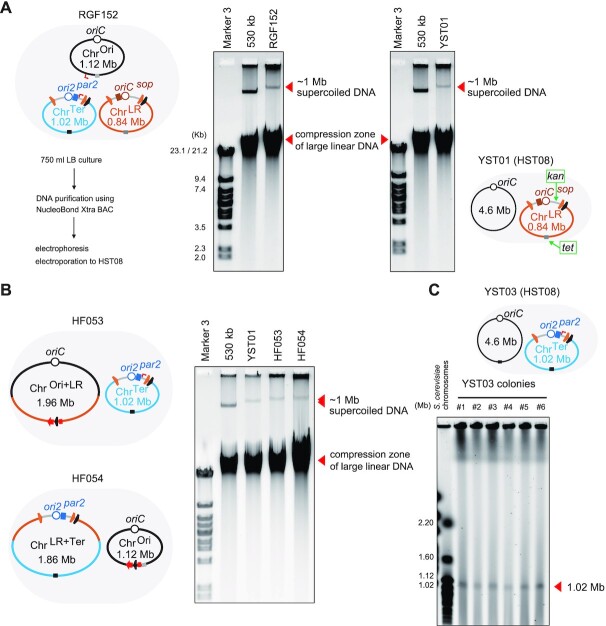
Purification and electroporation of 1-Mb chromosomes. (**A**) The split-chromosomes of RGF152 were purified with NucleoBond Xtra BAC kit, analysed by agarose gel electrophoresis, electroporated into HST08. A 127.5 ng aliquot and 4 × 51 ng aliquots of purified DNA (total 21 μg) were used for electrophoresis and electroporation, respectively. By using four vials of commercial electrocompetent cells of HST08, two positive colonies were obtained. The 0.84-Mb Chr^LR^ chromosome was purified from YST01, one of the Chr^LR^-transformed HST08 strains. The agarose gel electrophoresis analyses were performed using 0.5% agarose, 0.5× TBE, at 60 V for 65 min. The DNA size markers used were Marker 3 (λ/HindIII + λ/EcoRI digest mixture) and a 530-kb chromosome purified in the same manner. (**B**) The 1-Mb split-chromosomes of HF053 and HF054 were purified and analysed in the same manner as in (A). (**C**) PFGE analysis of the Chr^Ter^ chromosomes of six strains of YST03, HST08 transformed with the Chr^Ter^ chromosome purified in (B).

*Chr^LR^*. Chromosomes were extracted from RGF152 cells by using the NucleoBond Xtra BAC kit (Takara Bio) essentially according to the manufacturer instruction. To increase DNA concentration, DNA pellets from the isopropanol precipitation step were collected into a single tube and later dissolved with TE buffer. As a control and as a size marker, a 530-kb chromosome (32) was purified in the same manner. The supercoiled forms of extracted chromosomes were resolved by a conventional agarose gel electrophoresis method using a 0.5% agarose gel (32). In the gel, we observed a single high molecular weight band (>530 kb) for sample RGF152 (Figure 5A). This indicated that any or all of the three 1-Mb chromosomes (1.12, 0.84 and 1.02 Mb) were purified in the supercoiled form. Meanwhile, a significant proportion of DNA molecules sheared during the purification process were migrated in the gel to the compression zone of large linear DNA. The purified chromosomes were directly electroporated into *E. coli* HST08 Premium Electro-Cells (Takara Bio), typical commercial competent cells with a competency of >1 × 10^9^ transformants/μl pUC19 plasmid. Note that the Chr^Ori^ should confer resistance to Zeocin and Blasticidin S, the Chr^LR^ to kanamycin, spectinomycin, and tetracycline, and the Chr^Ter^ only to kanamycin. Ten colonies showed resistance to kanamycin and tetracycline among hundreds of kanamycin-resistance colonies. No Blasticidin S-resistant colony was obtained, indicating the absence of the Chr^Ori^. The colony-direct PCR analysis of six Kan^R^-Tet^R^ colonies and ninety-six Kan^R^ colonies suggested that three Kan^R^-Tet^R^ colonies might have a whole Chr^LR^. In contrast, none of the ninety-six colonies had a whole Chr^Ter^. By using the NucleoBond Xtra BAC kit, the whole Chr^LR^ was purified from cultures of one of the three Kan^R^-Tet^R^ strains, YST01 (Figure 5A), while the size of the Chr^LR^ chromosome of YST01 was confirmed by a PFGE analysis (data not shown). These results showed that the 0.84-Mb Chr^LR^ chromosome was purified and electroporated. It is likely that the placement of the *kan* and *tet* genes at the opposite poles of Chr^LR^ (Figure 5A) facilitated the selection of transformants.

*Chr^Ter^ and Chr^Ori^*. To purify Chr^Ter^ and Chr^Ori^ chromosomes individually, we used bipartite-genome strains rather than tripartite-genome strains. Bipartite-genome strains HF053 and HF054 were newly developed via Flp-POP cloning by using a smaller BAC vector pVtu9xF. HF053 has Chr^Ori+LR^ and Chr^Ter^, while HF054 has Chr^Ori^ and Chr^LR+Ter^. The 1.02-Mb Chr^Ter^ and the 1.12-Mb Chr^Ori^ purified from HF053 and HF054 cells, respectively, migrated slightly more slowly than the 0.84-Mb Chr^LR^ in an agarose gel on agarose gel electrophoresis (Figure 5B). Interestingly, the Chr^Ori+LR^ and Chr^LR+Ter^ were not co-purified with the Chr^Ter^ and Chr^Ori^, probably because the 1.86-Mb and 1.96-Mb chromosomes were too large and fragile. This result demonstrated that circular chromosomes of up to 1.1 Mb in size can be purified by using the NucleoBond Xtra BAC kit, which employs alkaline lysis method, anion exchange columns, and isopropanol precipitation. Next, HST08 competent cells were electroporated using the purified Chr^Ter^ and Chr^Ori^ chromosomes. Again, no colony contained the Chr^Ori^. On the other hand, 6 colonies among 110 Kan^R^ colonies were indicated to carry a whole Chr^Ter^ according to their colony direct PCR analysis. A PFGE analysis of cultures of the 6 colonies (or YST03 strains) confirmed the existence and the size of the whole Chr^Ter^ (Figure 5C). This result clearly proved that circular chromosomes of up to 1.0 Mb in size can be electroporated into *E. coli* competent cells.

### Partial genome swap via conjugation

**Figure 6.**
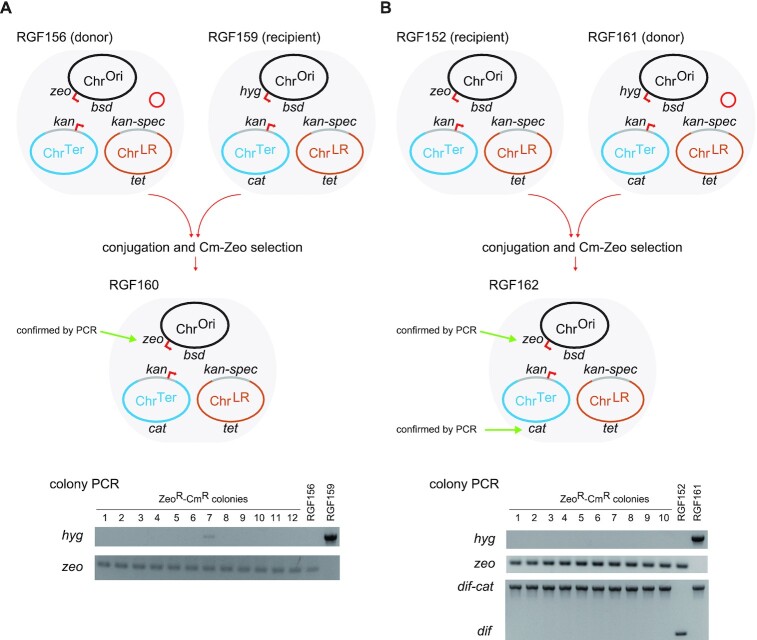
Partial genome swapping via conjugation. The donor strain carries a helper plasmid for conjugation, pBAD-traRP4min, indicated with a red circle. The Chr^Ori^ and Chr^Ter^ chromosomes contain an *oriT* sequence whose direction of transfer is indicated with red arrows. (**A**) The Chr^Ori^ of RGF159 was swapped by the Chr^Ori^ of RGF156 (RGF152 with the helper plasmid). Note that the *zeo* marker is the last gene transferred via conjugation from the *oriT* sequence. (**B**) The Chr^Ter^ of RGF152 was swapped by the Chr^Ter^ of RGF161 (RGF159 with the helper plasmid). The resultant strains RGF160 (A) and RGF162 (B) shared the same genotype. Selection marker gene loci were confirmed by colony PCR analyses as shown in the bottom panels (A, B).

The remaining question was whether the 1.1-Mb Chr^Ori^ was too large for electroporation or just unacceptable for the cell. It is very likely that the replication and partitioning systems of the 1.1-Mb Chr^Ori^ would interfere with that of the genome chromosome of the host cell and that the duplication of the highly-transcribed genetic loci such as the *rrnD* operon (42) would be a significant burden to the host cell. To assess this hypothesis, an extra Chr^Ori^ was transferred via *oriT*-mediated conjugation between tripartite-genome strains from RGF152 to RGF159 (Figure 6A). Remember that the Chr^Ori^ of RGF152 has the *zeo* gene, while the Chr^Ori^ of RGF159 has the *hyg* gene. The *zeo* gene is the last gene to be transferred upon conjugation. In addition, the Chr^Ter^ of the recipient strain RGF159 has the *cat* gene. After conjugation, no Zeo^R^-Hyg^R^-Cm^R^ colony was obtained, while Zeo^R^-Cm^R^ colonies were obtained. This indicated that the Chr^Ori^ of RGF152 was successfully transferred to RGF159 via conjugation and then replaced the original Chr^Ori^. On the other hand, preliminary attempts of the conjugal transfer of Chr^Ori^ to HST08-derived cells were not successful (data not shown), implying that the Chr^Ori^ may have been unacceptable for the wildtype *E. coli*. Furthermore, we performed the swapping of the Chr^Ter^ in RGF152 via conjugation by the Chr^Ter^ of RGF159 containing the *cat* gene (Figure 6B). The incompatibility between the two *ori2-par2* chromosomes may have driven the Chr^Ter^ swapping.

## DISCUSSION

We showed that once the chromosomes are down to 1 Mb in size they can be purified and directly electroporated into *E. coli* competent cells. It is likely that the physical compactness of the supercoiled 1-Mb chromosomes may have enabled their purification with columns and their transfer into *E. coli* cells via electroporation. On the other hand, large circular DNA molecules extracted in a traditional manner from cells digested in agarose plugs are often nicked/gapped and relaxed (32) (Fujita *et al.*, unpublished data). These findings may explain why *E. coli* had not shown such competency for genome-size DNA uptake. In other words, supercoiling of bacterial chromosomes is a key technology for synthetic biology and synthetic genomics. Previously, we have reconstituted the chromosome-replication cycle reaction (RCR) system of *E. coli* (32,43). The RCR system has been used for the *in vitro* amplification of *oriC*-containing chromosomes and for the supercoiling of circular chromosomes (32,43) (Fujita *et al.*, unpublished data). Thus, our methods may be applicable to circular chromosomes assembled *in vitro*, purified from yeast, or derived from other bacteria.

It was implied that the combination/choice of the BAC vector backbones affected the replication and partitioning of the split-chromosomes. RGF138 and its derivative strains were relatively healthy, whereas RGF140 cells became filamentous and sick. We assume that this is in part due to a difference in replication timing between the *ori2*- and *oriC*-BAC vectors. The initiation at *oriC* is regulated to occur synchronously in *E. coli* cells bearing multiple *oriC* sites (44), whereas the *ori2* initiation occurs after the *oriC* (*ori1*) initiation in the two-chromosome carrying *Vibrio* species (45,46). RGF138 carries the Ter region on the late replicating *ori2*-BAC vector, while the Ter region is on the early replicating *oriC*-BAC vector in RGF140. The earlier timing of the Ter region replication might disturb cell cycle and induce the filamentous and sick phenotype (45,47). It would be important to optimize the arrangement of genes and regulatory loci in the multipartite genome, because the copy numbers, intracellular locations, and local topologies of each genomic loci may be different from those in the wildtype *E. coli*.

There are many technological challenges towards multistep implantation of split-chromosomes into the same *E. coli* cells. The size and number of split-chromosomes should be reduced. It is ideal if each of the split-chromosomes could be easily transferred to cloning cells and stably maintained in the cells. For example, further engineering of the Chr^Ori^ chromosome, such as addition of a strong *par* system and elimination of a few ribosomal genes, would produce a more portable Chr^Ori^. Furthermore, the electroporation efficiency of chromosomes must be improved. Although we obtained 36 Chr^LR^-carrying colonies by using four vials of HST08 electrocompetent cells, we have not yet succeeded in the electroporation of any 1-Mb chromosome using self-made competent cells of a DGF-298-derived strain. Increasing the concentration and purity of the supercoiled form of chromosomes would help increase the efficiency.

## Supplementary Material

gkab298_Supplemental_Files
